# 

**Published:** 1876-10

**Authors:** 


### CONTENTS:

ARTICLE I.—Hygienic Laws. By G. W. Klump, D. D. S.... 241

ARTICLE II.—Dental College Education. By E. S. Talbot, D. D. S.... 250

ARTICLE III—Prevention and Treatment of Approximate Decay. By A. Warner, Jr., D. D S.... 257

ARTICLE IV.—Sarcomatous Tumors of the Jaws. By J. Ewing Mears, M. D.... 263

ARTICLE V.—Odontological Society of Great Britain.... 267

ARTICLE VI.—Treatment of Exposed Pulps by Lacto Phosphate of Lime. By Junius E. Cravens, D. D. S.... 274

ARTICLE VII.—Absorption of Dentine. By X. L.... 278

### EDITORIAL, ETC.

Correction.... 280
Wheat and Flour.... 280

### BIBLIOGRAPHICAL.

Wilkerson's Dental Register and Examiner.... 282
Micro-Photographs in Histology—Normal and Pathological.... 282

### MONTHLY SUMMARY.

The Comparative Effects of Ether and Chloroform.... 283
Caution in the Use of Chloral.... 284
Composition of the Human Body.... 285
The Blue Line in Lead Poisoning.... 286
Toothache Remedy.... 286
Women's Sphere.... 286
Nerve-Stretching in Tetanus.... 287
Progress of Cadaveric decay.... 288
Female Doctors.... 288

